# Autonomous Navigation System Using a Fuzzy Adaptive Nonlinear H∞ Filter

**DOI:** 10.3390/s140917600

**Published:** 2014-09-19

**Authors:** Fariz Outamazirt, Fu Li, Lin Yan, Abdelkrim Nemra

**Affiliations:** 1 School of Automation Science and Electrical Engineering, Beihang University, 37 Xueyuan Road, Haidian District, 100191 Beijing, China; E-Mails: fuli@buaa.edu.cn (F.L.); linyanee2@yahoo.com.cn (L.Y.); 2 School of Control and Automation, Ecole Militaire Polytechnique, EMP, Bordj El Bahri, 16111 Algiers, Algeria; E-Mail: karim_nemra@yahoo.fr

**Keywords:** UAV localization, sensor data fusion, Extended Kalman Filter (EKF), Nonlinear H∞ (NH∞), Fuzzy Adaptive Nonlinear H∞ (FANH∞) filter

## Abstract

Although nonlinear H∞ (NH∞) filters offer good performance without requiring assumptions concerning the characteristics of process and/or measurement noises, they still require additional tuning parameters that remain fixed and that need to be determined through trial and error. To address issues associated with NH∞ filters, a new SINS/GPS sensor fusion scheme known as the Fuzzy Adaptive Nonlinear H∞ (FANH∞) filter is proposed for the Unmanned Aerial Vehicle (UAV) localization problem. Based on a real-time Fuzzy Inference System (FIS), the FANH∞ filter continually adjusts the higher order of the Taylor development thorough adaptive bounds (*δ_i_*) and adaptive disturbance attenuation (*γ*), which significantly increases the UAV localization performance. The results obtained using the FANH∞ navigation filter are compared to the NH∞ navigation filter results and are validated using a 3D UAV flight scenario. The comparison proves the efficiency and robustness of the UAV localization process using the FANH∞ filter.

## 1. Introduction

Unmanned Aerial Vehicles (UAVs) are rapidly becoming a strategic asset in today's military forces and in the civilian airspace community. They can be used in an increasing number applications, such as surveillance, reconnaissance, communication relay, target designation and payload delivery [1]. The term UAV encompasses a wide variety of robotic aircrafts that vary in size, shape, flight characteristics and level of operational autonomy. The autonomy of these vehicles requires the development of navigation and guidance algorithms for self-localization. One important aspect of autonomous navigation, which should be investigated, is the fusion of data from different sensors. The Inertial Navigation System (INS) and the Strapdown Inertial Navigation System (SINS) in particular have been a vital source of navigation for UAVs [1]. The SINS is considered as a comprehensive navigation source because it is the only source that provides complete navigational information, such as position, velocity, and attitude, at a high data rate and with great precision on a short-term basis. However, the SINS's diverging errors caused by the integration process require an absolute sensor, such as a Global Positioning System (GPS), to constrain these drifts. To circumvent this limitation, one cost effective solution is to resort to an integrated navigation system in which the unboundedly growing trend of the errors of the SINS is contained by external navigation aids. The SINS has been augmented by different navigation aids over the last two decades, and GPS is the most prominent system used in integrated navigation systems among the systems that provide position and velocity fixes for the SINS.

The technology of multisensor data fusion is rapidly evolving in aerial navigation, where significant research has been devoted to developments concerning the UAV SINS/GPS localization problem in the last decade. One of the major concerns has been the issue of improving the accuracy, coverage, and reliability of the automatic navigation system within the imposed weight and cost constraints. The most mature technique used in navigation data fusion is the Kalman Filter (KF), which is a stochastic estimator that is typically used to solve various estimation problems and that is applied to a linear process and an observation model using a Gaussian statistical distribution of the process and observation noise. Various approximations have been developed in the literature, such as Extended Kalman Filters (EKFs), which is based on a first-order linearization of the nonlinear stochastic system models with the assumption of Gaussian distributed noises, to overcome the nonlinear filtering problems of integrated navigation systems. Although the EKF maintains the elegant and computationally efficient updated form of the KF, it suffers from a number of drawbacks [2]. If the filter is ill-conditioned due to modeling error, incorrect tuning of the covariance matrices, or initialization, and the subsequent estimation error will affect the linearization error. In turn, the latter will affect the estimation process and is known as a filter divergence. For this reason, the EKF requires greater care in modeling and tuning compared to the linear KF [3].

In this paper a robust alternative to the EKF that is based on NH∞ filtering to avoid the issues linked with modeling error and noise uncertainties is investigated to solve the UAV localization problem. The advantage of this filtering approach is that no assumptions are made regarding the statistical proprieties of the disturbance, and the filter is designed to minimize the estimation error due to the worst-case estimation error rather than the covariance of the estimation error [4]. In recent years, increased interest in the H∞ filter has led to several publications that address the H∞ nonlinear filter [5–10], where different approaches have been developed. However, in the current paper, we consider the approach used in studies [5,10] to construct a state estimator based on linearization for approximating the robust filtering known as the Robust Extended Kalman Filter (REKF), Extended H∞ Filter or Nonlinear H∞ filter. For the latter, the higher-order terms of the Taylor series expansion are not neglected but rather are assumed to be functions of the state estimation error and of the exogenous inputs, which have bounded H∞ norms and which lead to a min-max estimation that can be treated using standard H∞ filter methods [5].

It should be mentioned that although the nonlinear H∞ filter offers a good performance without presuming characteristics of the process and/or measurement noises, the filter still requires additional tuning parameters that remain fixed and that need to be determined using trial and error [3]. These parameters may be used to control the compromise between the two performance criteria and the scaling of the inputs to accommodate linearization errors [5]. Motivated by this issue, the Adaptive Robust Extended Kalman filter for a nonlinear system was proposed in [11], where the primary goal was to design an estimator based on stability analyses and to determine if the error covariance matrix should be reset based on the hypothesis test [11]. It should be noted that only the tuning of the disturbance attenuation (*γ*) has been considered in the filtering process, and the higher order terms of the Taylor development have been neglected when calculating the Jacobian. The results of the current paper are thus complementary to the results obtained in [3]. In this paper we extend the nonlinear H∞ (NH∞) filter to include a fuzzy adaptive scheme.

To address the issues associated with the NH∞ filter, a new SINS/GPS sensor fusion scheme known as the Fuzzy Adaptive Nonlinear H∞ (FANH∞) filter for the UAV localization problem is proposed in this study. Based on a real-time Fuzzy Inference System (FIS), the FANH∞ filter continually adjusts the higher order of the Taylor development thorough the adaptive bounds (*δ_i_*) as well as the adaptive disturbance attenuation (*γ*), which significantly increases the UAV localization performance. The results obtained by the FANH∞ navigation filter were compared to the NH∞ navigation filter results and were validated using a 3D UAV flight scenario. The comparison proves the efficiency and robustness of the UAV localization process using the FANH∞ filter.

The remainder of this paper is organized as follows. The sensor model for the SINS and the GPS is discussed in Section 2. A brief overview of the EKF algorithm is stated in Section 3. Section 4 contains the formulation of the Nonlinear H∞ (NH∞) filter and its drawbacks. Section 5 is devoted to the Fuzzy Adaptive Nonlinear H∞ (FANH∞) filter. Lastly, the simulation results are provided to illustrate the performance of the FANH∞ filter for the UAV localization problem and are compared with the Nonlinear H∞ (NH∞) filter.

## 2. Mathematical Model of Integrated Navigation System for SINS/GPS

### 2.1. References Frame

**Figure 1.**
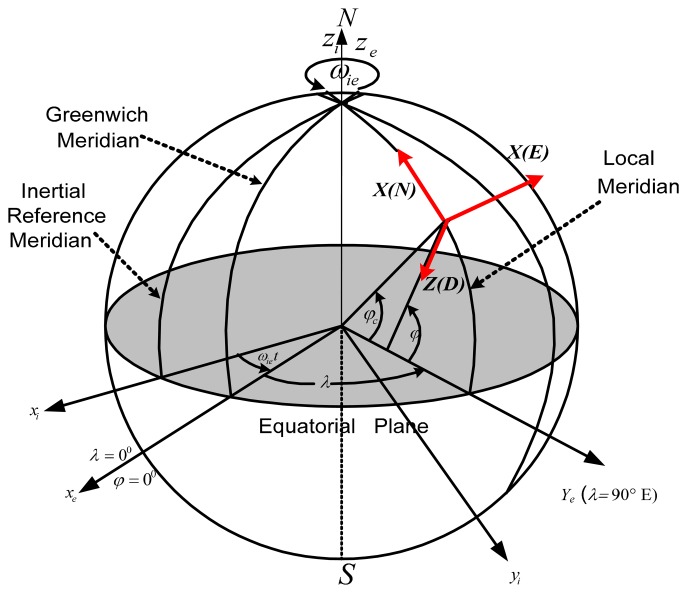
3D reference frame geometry.

An important part of the inertial navigation system analysis consists of determining the relation between the different frames. The body frame is the basic frame for the inertial sensor: the *x*-axis is pointing forward, the *y*-axis is pointing to the right, and the z-axis completes the right-hand orthogonal system by pointing downwards. The North-East-Down (NED) is the navigation frame: the *N* vector is pointing North, the *E* vector is pointing East, and the *D* vector is pointing Down along the local gravity vector, as indicated in Figure 1 [12].

The Inertial Measurement Unit (IMU) measures the acceleration (*ax*,*ay*,*az*) and the rotation rate (*p*,*q*,*r*) high update rate [13]. These vectors are transformed to the navigation frame. The transformation matrix used is the Direct Cosine Matrix *C_bn_*, which represents the attitude of the body frame with respect to the navigation frame and can be expressed in terms of three rotational Euler angles R(*ϕ*), *R*(*θ*) and *R*(*ψ*), which signify the roll, pitch and yaw, respectively, as follows [12,13]:

(1) $$C_{bn}=R(\phi ,\theta ,\psi )=R(\phi )R(\theta )R(\psi )$$

(2) $$C_{bn}=\left[\begin{array}{ccc} 1 & 0 & 0\\ 0 & \cos\phi & \sin\phi \\ 0 & -\sin\phi & \cos\phi \end{array}\right]\;\left[\begin{array}{ccc} \cos\theta & 0 & -\sin\theta \\ 0 & 1 & 0\\ \sin\theta & 0 & \cos\theta \end{array}\right]\;\left[\begin{array}{ccc} \cos\psi & \sin\psi & 0\\ -\sin\psi & \cos\psi & 0\\ 0 & 0 & 1 \end{array}\right]$$

(3) $$C_{bn}=\left[\begin{array}{ccc} \cos(\theta )\cos(\psi ) & \cos(\theta )\sin(\psi ) & -\sin(\theta )\\ \sin(\theta )\sin(\phi )\cos(\psi )-\sin(\psi )\cos(\phi ) & \sin(\psi )\sin(\theta )\sin(\phi )+\cos(\psi )\cos(\phi ) & \sin(\phi )\cos(\theta )\\ \sin(\theta )\cos(\phi )\cos(\psi )+\sin(\psi )\sin(\phi ) & \sin(\phi )\sin(\theta )\cos(\phi )-\cos(\psi )\sin(\phi ) & \cos(\phi )\cos(\theta ) \end{array}\right]$$

### 2.2. Equation of Motion

We can transform the rotation rates (*p*,*q*,*r*) from the body frame to the navigation frame to calculate the Euler angle rates (*ϕ˙*,*θ˙*,*ψ˙*) as follows:

(4) $$\left[\begin{array}{c} p\\ q\\ r \end{array}\right]=\left[\begin{array}{c} \dot{\phi }\\ 0\\ 0 \end{array}\right]+R(\phi )\;\left[\begin{array}{c} 0\\ \dot{\theta }\\ 0 \end{array}\right]+R(\phi )R(\theta )\;\left[\begin{array}{c} 0\\ 0\\ \dot{\psi } \end{array}\right]$$

Then, the equation can be written as follows:

(5) $$\left[\begin{array}{c} p\\ q\\ r \end{array}\right]=\left[\begin{array}{ccc} 1 & -\sin(\theta ) & 0\\ 0 & \cos(\theta ) & \cos(\theta )\sin(\phi )\\ -\sin(\phi ) & 0 & \cos(\theta )\cos(\phi ) \end{array}\right]\;\left[\begin{array}{c} \dot{\phi }\\ \dot{\theta }\\ \dot{\psi } \end{array}\right]$$

(6) $$\left[\begin{array}{c} p\\ q\\ r \end{array}\right]=C_{\left(p,q,r/\dot{\phi },\dot{\theta },\dot{\psi }\right)}\;\left[\begin{array}{c} \dot{\phi }\\ \dot{\theta }\\ \dot{\psi } \end{array}\right]$$

To solve (*ϕ̇*,*θ̇*,*ψ̇*)*^T^*, we can calculate $C_{\left(p,q,r/\dot{\phi },\dot{\theta },\dot{\psi }\right)}^{-1}$. Then, the Euler angle rates can be expressed as follows:

(7) $$\left[\begin{array}{c} \dot{\phi }\\ \dot{\theta }\\ \dot{\psi } \end{array}\right]=\left[\begin{array}{ccc} 1 & \sin(\phi )\tan(\theta ) & \cos(\phi )\tan(\theta )\\ 0 & \cos(\phi ) & -\sin(\phi )\\ 0 & \sin(\phi )\sec(\theta ) & \cos(\phi )\sec(\theta ) \end{array}\right]\;\left[\begin{array}{c} p\\ q\\ r \end{array}\right]$$

### 2.3. Navigation Equations

We assume that the IMU is at the vehicle's center of gravity, and to solve for the vehicle acceleration, we must subtract the known gravity component from the measured accelerations [12,13]. The true vehicle acceleration (*U̇*,*V̇*,*Ẇ*) in the body frame can be expressed as follows:

(8) $$\left[\begin{array}{c} \dot{U}\\ \dot{V}\\ \dot{W} \end{array}\right]=\left[\begin{array}{c} U\\ V\\ W \end{array}\right]\times \left[\begin{array}{c} p\\ q\\ r \end{array}\right]+\left[\begin{array}{c} ax\\ ay\\ az \end{array}\right]-\left[\begin{array}{c} g_{x}\\ g_{y}\\ g_{z} \end{array}\right]$$

(9) $$\left[\begin{array}{c} \dot{U}\\ \dot{V}\\ \dot{W} \end{array}\right]=\left[\begin{array}{ccc} 0 & -W & V\\ W & 0 & -U\\ -V & U & 0 \end{array}\right]\;\left[\begin{array}{c} p\\ q\\ r \end{array}\right]+\left[\begin{array}{c} ax\\ ay\\ az \end{array}\right]-\left[\begin{array}{c} g_{x}\\ g_{y}\\ g_{z} \end{array}\right]$$

where (*ax*,*ay*,*az*) are the measured accelerations in the body frame, and (*g_x_*,*g_y_*,*g_z_*) are the gravity components expressed in the body frame as follows:

(10) $$\left[\begin{array}{c} g_{x}\\ g_{y}\\ g_{z} \end{array}\right]=C_{bn}\;{\left[\begin{array}{ccc} 0 & 0 & g_{e} \end{array}\right]}^{T}=\left[\begin{array}{c} -g_{e}\sin(\theta )\\ g_{e}\cos(\theta )\sin(\phi )\\ g_{e}\cos(\theta )\cos(\phi ) \end{array}\right]$$

By substituting Equation (10) into Equation (9), the true vehicle acceleration (*U̇*,*V̇*,*Ẇ*) in the body frame [12,13] can be expressed as follows:

(11) $$\left[\begin{array}{c} \dot{U}\\ \dot{V}\\ \dot{W} \end{array}\right]=\left[\begin{array}{c} ax+Vr-Wq+g_{e}\sin(\theta )\\ ay-Ur+Wp-g_{e}\cos(\theta )\sin(\phi )\\ az+Uq-Vp-g_{e}\cos(\theta )\cos(\phi ) \end{array}\right]$$

The resulting acceleration vector is integrated with respect to time to obtain the velocity of the vehicle (*U*,*V*,*W*) in the body frame [12,13] as follows:

(12) $$\left[\begin{array}{c} U\\ V\\ W \end{array}\right]=\int \left[\begin{array}{c} \dot{U}\\ \dot{V}\\ \dot{W} \end{array}\right]dt$$

The resulting velocity vector is then integrated to obtain the position of the vehicle. If the velocity is transformed to the navigation frame and integrated, we can obtain the position of a vector [*X*,*Y*,*Z*]*^T^* as follows:

(13) $$\left[\begin{array}{c} X\\ Y\\ Z \end{array}\right]=\int {C_{bn}}^{T}(\phi ,\theta ,\phi )\;\left[\begin{array}{c} U\\ V\\ W \end{array}\right]dt$$

### 2.4. Nonlinear Model Using Euler Angles

The nonlinear model of the SINS can be defined as follows:

(14) $$\left\{ \begin{array}{l} \dot{x}(t)=f(x(t),u(t),t)\\ y(t)=h(x(t),u(t),t) \end{array}\right.$$

where *x* is the state vector, which contains the position, velocity, Euler angles, constant random drifts in the gyros and constant random biases in the accelerometers as follows:

(15) $$x={\left[\begin{array}{ccccccccccccccc} X & Y & Z & U & V & W & \phi & \theta & \psi & \varepsilon_{bx} & \varepsilon_{by} & \varepsilon_{bz} & \nabla_{bx} & \nabla_{by} & \nabla_{bz} \end{array}\right]}^{T}$$

where *ε_bx_*, *ε_by_* and *ε_bz_* are the constant random drifts in the gyros and ∇*_bx_*, ∇*_by_* and ∇*_bz_* are the constant random biases in the accelerometers.

Thus, *u* represents the IMU outputs, where the angles' rate and accelerations can be expressed as follows:

(16) u=[p,q,r,ax,ay,az]

We can place Equations (13), (11) and (7) into the matrix, which provides the (15×15) state transition matrix to present the nonlinear model that describes the air vehicle [12]. The nonlinear SINS state model can be written as follows:

(17) $$f(x,u)=\left[\begin{array}{l} {\left[\begin{array}{ccc} \cos(\theta )\cos(\psi ) & \cos(\theta )\sin(\psi ) & -\sin(\theta )\\ \sin(\theta )\sin(\phi )\cos(\psi )-\sin(\psi )\cos(\phi ) & \sin(\psi )\sin(\theta )\sin(\phi )+\cos(\psi )\cos(\phi ) & \sin(\phi )\cos(\theta )\\ \sin(\theta )\cos(\phi )\cos(\psi )+\sin(\psi )\sin(\phi ) & \sin(\phi )\sin(\theta )\cos(\phi )-\cos(\psi )\sin(\phi ) & \cos(\phi )\cos(\theta ) \end{array}\right]}^{T}\left[\begin{array}{c} U\\ V\\ W \end{array}\right]\\ \left[\begin{array}{c} ax+Vr-Wq+g_{e}\sin(\theta )\\ ay-Ur+Wp-g_{e}\cos(\theta )\sin(\phi )\\ az-Uq-Vp-g_{e}\cos(\theta )\cos(\phi ) \end{array}\right]\\ \left[\begin{array}{ccc} 1 & \sin(\phi )\tan(\theta ) & \cos(\phi )\tan(\theta )\\ 0 & \cos(\phi ) & -\sin(\phi )\\ 0 & \sin(\phi )\sec(\theta ) & \cos(\phi )\sec(\theta ) \end{array}\right]\;\left[\begin{array}{c} p\\ q\\ r \end{array}\right]\\ \left[\begin{array}{c} 0\\ 0\\ 0 \end{array}\right]\\ \left[\begin{array}{c} 0\\ 0\\ 0 \end{array}\right] \end{array}\right]$$

**Figure 2.**
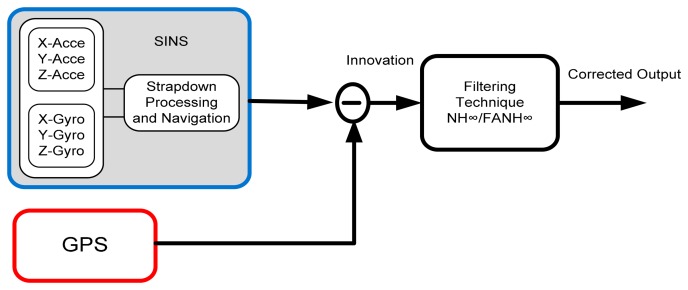
Aided SINS/GPS system configuration.

The SINS, which is equipped with high-rate sensors (gyros and accelerometers), is a self-contained navigation system; it provides high precision and accuracy for shorter durations of time and is immune from external effects, such as jamming. However, during navigation, the SINS errors accumulate and increase, which causes the SINS to be unsuitable for long-term navigation [14,15]. The GPS can provide accurate, real-time three-dimensional position and velocity information with only random errors, which do not increase unboundedly. The powerful synergy between the GPS and SINS is illustrated in Figure 2.

This synergy is possible, in part, because both systems have extremely complementary error characteristics. The short-term position errors from the SINS are relatively small, but they degrade without bound over time. Conversely, the GPS position errors are not as suitable over the short term, but they do not degrade with time [16].

An essential component in a satellite navigation system, e.g., the GPS, is the availability of satellites correctly transmitting coded signals from known positions. Three satellites are required to provide three distance measurements, while a fourth satellite is required to remove receiver clock error. The technique used is trilateration based on the geometry of circles, where an unknown point can be calculated from three known points. The intersection of the arc corresponding to the three distances defines the unknown point relative to the known points. The three measurements can be used to solve the three equations to determine the longitude, latitude and elevation of the receiver [17]. The calculation using four satellites provides the receiver with sufficient information to calculate its position with high accuracy. Four satellites, rather than three, are required because the clock in the receiver is not accurate enough. The fourth distance measurement provides information from which the clock errors in the receiver can be corrected, and the receiver clock is synchronized to the GPS with an accuracy of greater than 100 ns.

The GPS satellites transmit two signals at different frequencies: *L*_1_ at *f*_1_ = 1575.42 MHz and *L*_2_ at *f*_2_ = 1227.6 MHz. The GPS system provides two categories of service. The Precise Positioning Service (PPS) receivers track both *P* codes on *L*_1_ and *L*_2_ frequencies. The PPS is used primarily by military users because the *P* code is encrypted into the *Y* code before transmission and requires decryption equipment in the receiver. The Standards Positioning Service (SPS) receivers track the *C*/*A* (Clear Acquisition) code on *L*_1_ [16,17].

The measurement model, which is related to the position and velocity of the UAV used in this paper can be expressed as follows:

(18) $$h(x,u)=\left[\begin{array}{ccc} I^{3x3} & 0^{3x3} & 0^{3x9}\\ 0^{3x3} & I^{3x3} & 0^{3x9} \end{array}\right]\;x$$

where [*X Y Z U V W ϕ θ ψ ε_bx_ ε_by_ ε_bz_* ∇_*bx*_ ∇_*by*_ ∇_*bz*_]*^T^*.

## 3. Extended Kalman Filter

Most real world systems, such as navigation systems, are nonlinear, and the standard Kalman filter cannot be used. To overcome this limitation, the Extended Kalman Filter (EKF) approximates the nonlinear system using the Jacobian to calculate the covariance of a random vector propagating through the nonlinear model [2,18,19]. The zero-order hold (ZOH) is used in this paper to convert the system defined in Equation (14) to a nonlinear discrete-time state transition equation. The system and the measurement equations can be written in a discrete form as follows [20]:

(19) $$x_{k}=f_{k-1}(x_{k-1},u_{k-1},w_{k-1})$$

(20) $$y_{k}=h_{k}(x_{k},v_{k})$$

where *w_k_* and *v_k_* are the uncorrelated zero mean white noise process with the known covariance *Q_k_* and *R_k_*, respectively.

The EKF approximate nonlinear system dynamics and measurement vehicle models uses the Jacobian to calculate the covariance of a random vector propagating through the nonlinear models. The nonlinear model and the measurement model expanded around the filtered and predicted estimates of *x̂_k_* and *x̂_k_*_−1_ can be defined as follows:

(21) $$x_{k}=f({\hat{x}}_{k-1/k-1},u_{k-1})+\Delta f_{k}(x)[x_{k}-{\hat{x}}_{k/k}]+\Delta_{1}(x_{k}-{\hat{x}}_{k/k})+[\Delta f_{w}(x)+\Delta_{2}(x_{k}-{\hat{x}}_{k/k})]w_{k}$$

(22) $$y_{k}=h({\hat{x}}_{k/k-1},u_{k})+\Delta h_{k}(x)[x_{k}-{\hat{x}}_{k/k-1}]+\Delta_{3}(x_{k}-{\hat{x}}_{k/k-1})+\nu_{k}$$

where Δ*f_k_*(*x*) is the Jacobian of *f* evaluated at *x_k_*_−1_, the filter state error *x̃_k/k_*_−1_ = *x_k_* − *x̃_k/k_*_−1_, the predictor state error *x̃_k/k_*_−1_ = *x_k_* − *x̃_k/k_*_−1_, Δ*f_w_*(*x*) is the Jacobian of (*f*/*w_k_*) evaluated at *x_k_*_−1_, Δ*h_k_*(*x*) is the Jacobian of *h* evaluated at *x_k_*_−1_, and Δ*_i_*_=__1,..3_ represents the higher-order terms of the Taylor series norm, which are bounded as ∥Δ*_i_*∥ ≤ *δ_i_* [13,20]. The reformulated state and measurement model after neglecting the higher-order terms of the Taylor series can be expressed as follows [13]:

(23) $$x_{k+1}=F_{k}x_{k}+B_{k}w_{k}$$

(24) $$y_{k}=H_{k}x_{k}+\nu_{k}$$

where *F_k_* = Δ*f_k_*(*x̃_k/k_*) and *B_k_* = Δ*f_w_*(*x̃_k/k_*).

The time update of the estimation error covariance can be defined as follows:

(25) $$x_{k+1/k}=f(x_{k/k},u_{k},0)$$

(26) $$P_{k+1/k}=F_{k}P_{k/k}F_{k}^{T}+B_{k}Q_{k}B_{k}^{T}$$

The measurement update of the state estimate and the estimation-error covariance can be defined as follows [20]:

(27) $$K_{k}=P_{k/k-1}H_{k}^{T}{\left(H_{k}P_{k/k-1}H_{k}^{T}+R_{k}\right)}^{-1}$$

(28) $${\hat{x}}_{k/k}={\hat{x}}_{k/k-1}+K_{k}[y_{k}-h_{k}({\hat{x}}_{k/k-1})]$$

(29) $$\begin{array}{c} P_{k/k}=P_{k/k-1}-P_{k/k-1}H_{k}^{T}{\left(H_{k}P_{k/k-1}H_{k}^{T}+R_{k}\right)}^{-1}H_{k}P_{k/k-1}\\ =(I-K_{k}H_{k})P_{k/k-1} \end{array}$$

The EKF has been typically used in several applications; however, its implementation assumes that the process and measurement models are known. When there are large deviations in the estimated system state trajectory, the nonlinear model in Equations (19) and (20) is weakly approximated by the Taylor series expansion around the conditional mean, hence the need for the higher-order terms of the Taylor expansion [3,5].

## 4. Nonlinear H∞ Fitler

The robust filters take different forms depending on the type of disturbances that are considered [11], whereas the common performance criterion of the filter is to ensure a bounded energy gain from the worst possible disturbance to the estimation error. The structure of the nonlinear H∞ algorithm used in this paper is the same as that developed in [5] and is proposed to solve the SINS/GPS UAV localization problem in [3]. The NH∞ filter is used to estimate the nonlinear model given in Equation (14) and satisfy the H∞ performance criterion for all uncertainties Δ_1_, Δ_1_ and Δ_3_ that are defined in Equations (21) and (22) and their norm bound [5]. Instead of the system defined in Equations (21) and (22), we consider the system defined as follows:

(30) $$x_{k+1}=F_{k}x_{k}+B_{k}w_{k}+{\text{M}}_{k}+{\text{T}}_{k}$$

(31) $$y_{k}=H_{k}x_{k}+\nu_{k}+\xi_{k}+\phi_{k}$$

(32) $${\tilde{x}}_{k/k}=x_{k}-{\hat{x}}_{k/k}$$

where T*_k_* = Δ_1_(*x̃_k/k_*) + Δ_2_(*x̃_k/k_*)*w_k_* and *ϕ_k_* = Δ_3_(*x̃_k/k_*_−1_) are extra exogenous inputs [5] fulfilling the following conditions:

(33) $$\left\|{\text{T}}_{k}\right\|\leq \delta_{1}^{2}{\left\|{\tilde{x}}_{k/k}\right\|}_{2}^{2}+\delta_{2}^{2}{\left\|w_{k}\right\|}_{2}^{2}$$

(34) $${\left\|\phi_{1}\right\|}_{2}^{2}\leq \delta_{3}^{2}{\left\|{\tilde{x}}_{k/k}\right\|}_{2}^{2}$$

(35) $${\text{M}}_{k}=f_{k}({\hat{x}}_{k/k})-F_{k}{\hat{x}}_{k/k}$$

(36) $$\xi_{k}=h_{k}({\hat{x}}_{k/k-1})-H_{k}{\hat{x}}_{k/k-1}$$

We can rewrite Equations (30) and (31), which contain the extra terms T*_k_* and *ϕ_k_* not used in the EKF, by scaling *w* and *v* [5,10] as follows:

(37) $$x_{k+1}=F_{k}x_{k}+B_{k}c_{w}w_{k}+{\text{M}}_{k}$$

(38) $$y_{k}=H_{k}x_{k}+c_{v}\nu_{k}+\xi_{k}$$

where:

(39) $$c_{v}^{2}=1-\gamma^{2}\delta_{1}^{2}-\gamma^{2}\delta_{3}^{2}$$

(40) $$c_{w}^{2}=c_{v}^{2}{\left(1+\delta_{2}^{2}\right)}^{-1}$$

The NH∞ has the structure of the EKF defined in Equation (25) to Equation (29), with the exception of the approximate error covariance correction (29), which can be substituted with:

(41) $$P_{k/k}=P_{k/k-1}-P_{k/k-1}[-C_{k}^{T}{\text{H}}_{k}^{T}]{\left[\begin{array}{cc} C_{k}P_{k/k-1}{\text{C}}_{k}^{T}-\gamma^{2}I & -C_{k}P_{k/k-1}{\text{H}}_{k}^{T}\\ -H_{k}P_{k/k-1}{\text{C}}_{k}^{T} & H_{k}P_{k/k-1}{\text{H}}_{k}^{T}+R_{k} \end{array}\right]}^{-1}\left[\begin{array}{c} -C_{k}\\ H_{k} \end{array}\right]P_{k/k-1}$$

where:

- *C_k_* = *I*; and
- *w_k_* and *ν_k_* are scaled using *c_w_* and *c_v_*, respectively.

The extended H∞ filter reverts to the EKF when the state error (*x_k_* − *x̂_k/k_*) and the process noise are extremely small; furthermore, *γ* → ∞ [5].

## 5. Fuzzy Adaptive Nonlinear H∞ Filter

**Figure 3.**
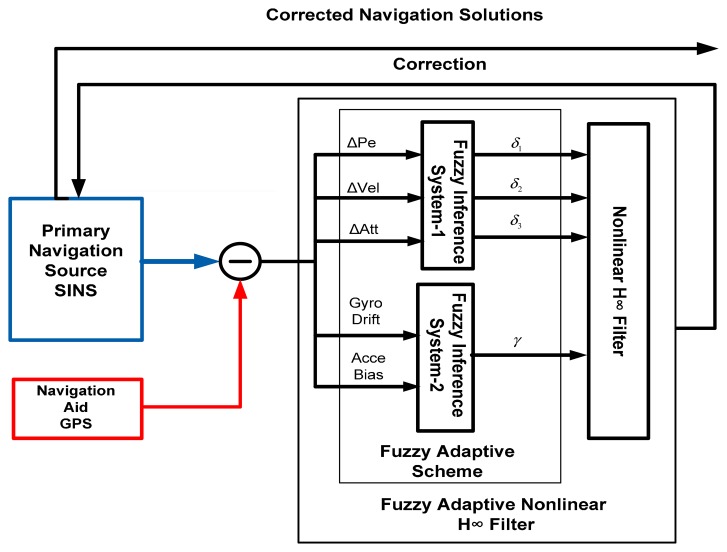
SINS/GPS integration based on a Fuzzy Adaptive Nonlinear H∞ filter.

By referring to [3,5,6,10], the parameters of the NH∞ filter remain fixed during their processing. The equations defined in Equations (37) and (38) have parameters *δ*_1_, *δ*_2_, *δ*_3_ and *γ* that can be adjusted and adapted in response to the parameters' uncertainty and change in environment. A new concept regarding the SINS/GPS integration based on the Fuzzy Adaptive Nonlinear H∞ (FANH∞) filters for the UAV localization problem is investigated in this paper. The adaptive approach based on the Fuzzy Inference System (FIS) is suggested to automatically tune the parameters of the NH∞ (*δ*_1_, *δ*_2_, *δ*_3_ and *γ*) filter. The FANH∞ filter continually adjusts the higher-order terms of the Taylor development thorough the adaptive bounds (*δ*_1_, *δ*_2_, *δ*_3_) as well as through the adaptive disturbance attenuation *γ*, which significantly increases the UAV localization performance. Online tuning using the FIS offers robust behavior without decreasing the accuracy and guarantees the boundedness of the estimator error even with the unknown disturbance and the linearization error. Thus, the proposed study consists of adjusting the four parameters *δ*_1_, *δ*_2_, *δ*_3_ and *γ* of the NH∞ filter using two Fuzzy Inference Systems. The developed FIS is illustrated in Figure 3 and consists of two fuzzy controllers known as FIS-1 and FIS-2 operating separately.

The first Fuzzy Inference System (FIS-1) has three inputs, position error (ΔPe), velocity error (ΔVel) and attitude error (ΔAtt), that represent the linearization errors that can be determined by subtracting the linearized nonlinear state model from the linearized nonlinear state model. The outputs of the first FIS-1 are *δ*_1_, *δ*_2_ and *δ*_3_.

Furthermore, the estimation environment in the case of the SINS/GPS kinematic applications is subject to change in the Gyroscope Drift (Gyro Drift) and in the Accelerometer Bias (Acce Bias), which represent the two inputs of the second FIS-2 that are provided as the output *γ*.

The output of the two fuzzy controllers primarily depends on the membership function and on the definition of the fuzzy rules. The input variables of the first FIS, *i.e.*, the position error (ΔPe), velocity error (ΔVel) and attitude error (ΔAtt), are divided into six triangular fuzzy sets, while the second FIS has two inputs, the accelerometer bias (Acce Bias) and the gyroscope bias (Gyro Bias), where each input is divided into four triangular fuzzy sets. The accelerometer and gyroscope bias are estimated by the FANH∞ filter simultaneously.

The FIS-1 control rules can be presented as follows:

1-IF (ΔPe is P) AND (ΔVel is P) AND (ΔAtt is P) THEN (*δ*_1_ is P) (*δ*_2_ is P) (*δ*_3_ is P).
2-IF (ΔPe is P) AND (ΔVel is P) AND (ΔAtt is G) THEN (*δ*_1_ is P) (*δ*_2_ is M) (*δ*_3_ is M).
3-IF (ΔPe is P) AND (ΔVel is G) AND (ΔAtt is P) THEN (*δ*_1_ is P) (*δ*_2_ is G) (*δ*_3_ is M).
4-IF (ΔPe is P) AND (ΔVel is G) AND (ΔAtt is G) THEN (*δ*_1_ is P) (*δ*_2_ is G) (*δ*_3_ is G).
5-IF (ΔPe is G) AND (ΔVel is P) AND (ΔAtt is P) THEN (*δ*_1_ is P) (*δ*_2_ is G) (*δ*_3_ is P).
6-IF (ΔPe is G) AND (ΔVel is G) AND (ΔAtt is G) THEN (*δ*_1_ is M) (*δ*_2_ is M) (*δ*_3_ is M).

The FIS-2 control rules can be presented as follows:

1-IF (Gyro Drift is P) AND (Acce Bias is P) THEN (*γ* is G).
2-IF (Gyro Drift is G) AND (Acce Bias is P) THEN (*γ* is M).
3-IF (Gyro Drift is G) AND (Acce Bias is G) THEN (*γ* is P).
4-IF (Gyro Drift is P) AND (Acce Bias is G) THEN (*γ* is M).

## 6. Simulation and Discussion of Results

We present our simulation results to validate the proposed Fuzzy Adaptive Nonlinear H∞ filter (FANH∞) for the Unmanned Aerial Vehicle localization problem. The results of our approach are compared with other navigation filtering approaches. The sampling rates used for each sensor and the update rate of the filters used in this study can be stated as follows:

$$f_{\text{SINS}}=100\text{Hz},f_{\text{EKF}}=10\text{Hz},f_{NH\infty }=10\text{Hz},f_{\text{GPS}}=1\text{Hz},f_{\text{FANS}\infty }=10\text{Hz}.$$

**Figure 4.**
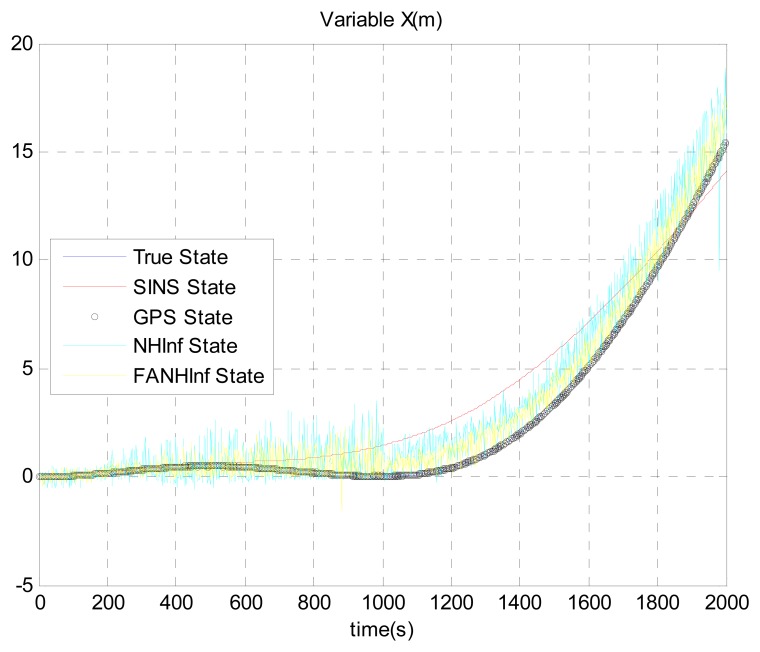
Estimation of the position *X*.

**Figure 5.**
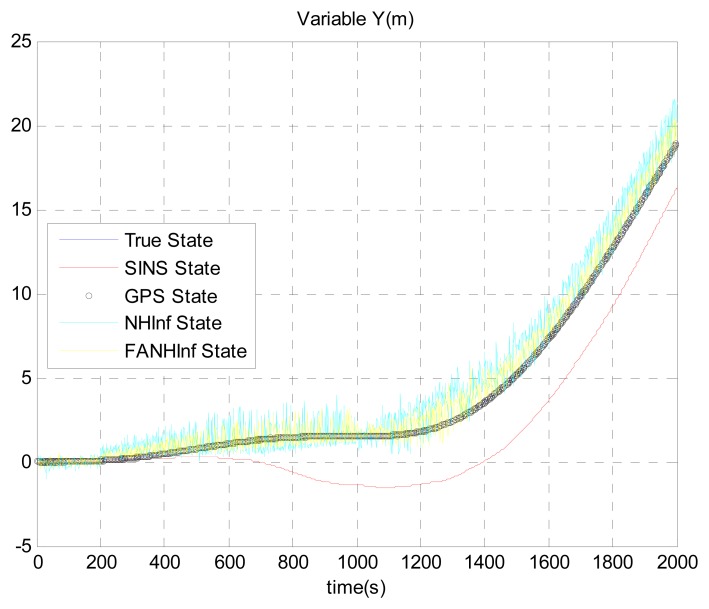
Estimation of the position *Y*.

**Figure 6.**
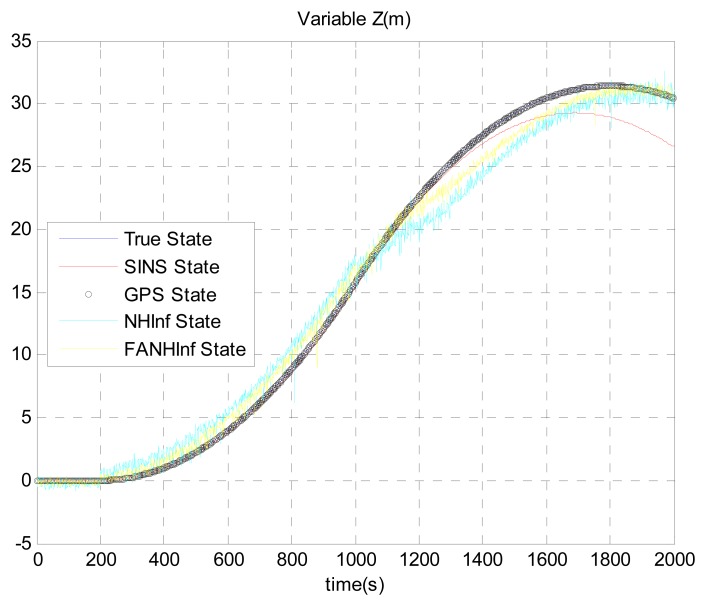
Estimation of the position *Z*.

**Figure 7.**
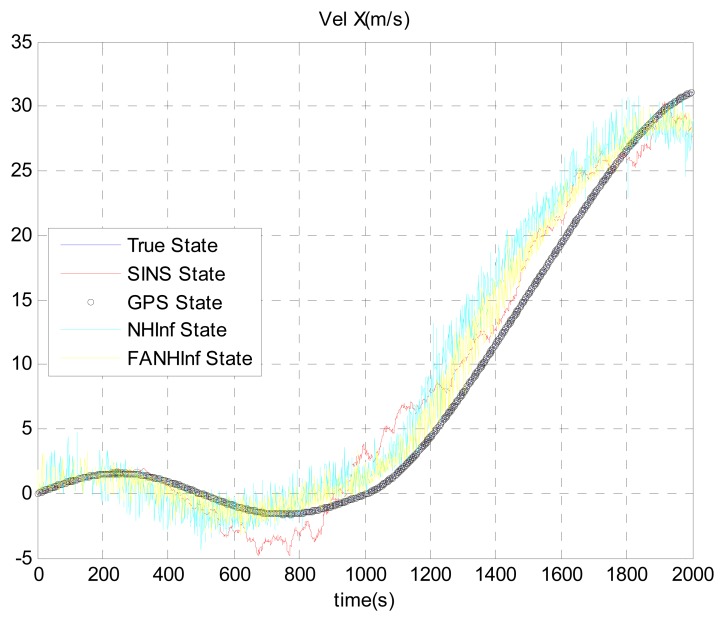
Estimation of the velocity following the North axis.

**Figure 8.**
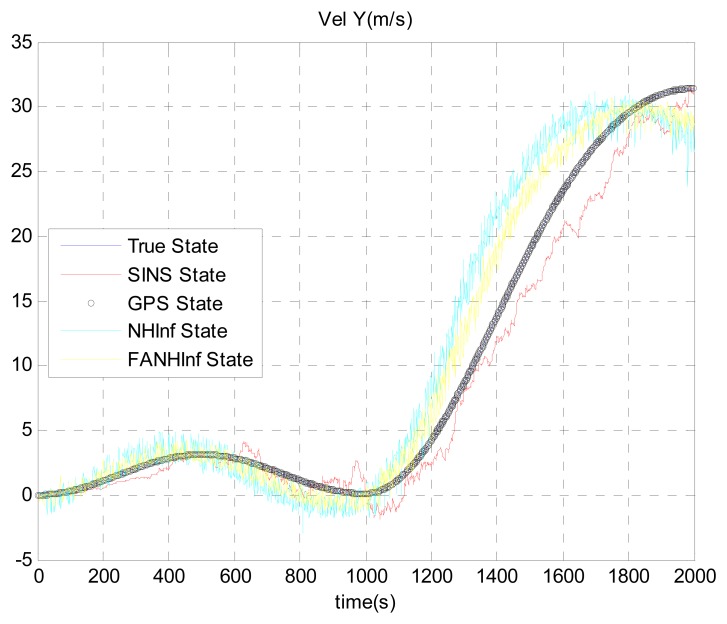
Estimation of the velocity following the East axis.

**Figure 9.**
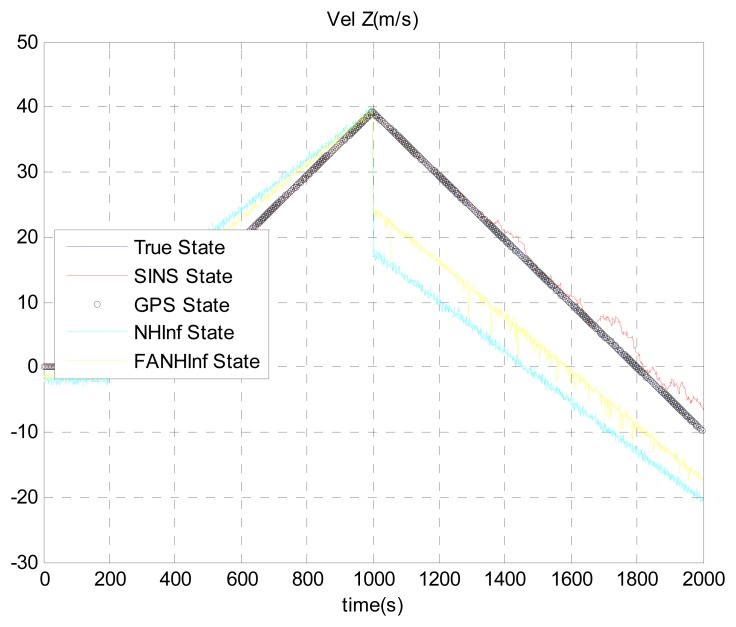
Estimation of the velocity following the Down axis.

The simulation results provided in Figures 4, 5 and 6 represent the estimated UAV position obtained using the SINS position, the NH∞ filter and the FANH∞ filter, respectively, in the *x*-, *y*- and *z*-axes. As seen from these figures, the performance of the FANH∞ filter is significantly greater than that of the NH∞ filter (bounds are predefined), which confirms the efficiency of the adaptive tuning of the NH∞ filter bounds. Table 1 presents and compares the average of 100 groups of standard deviations in the *x*-, *y*- and *z*-axes with the EKF, NH∞ and FANH∞ filters. It is evident from the table that the proposed filter provides a more accurate position without any pre-assumption of the characteristics of the process and measurement noises or the H∞ bounds. The automatic tuning of the NH∞ filter bound significantly reduces the accuracy of the position estimation. Similar results have been obtained for the velocity estimation. From Figures 7, 8 and 9, we can observe the benefit of using fuzzy adaptive tuning for the NH∞ bounds (*δ*_1_, *δ*_2_, *δ*_3_ and *γ*).

Table 2 provides a comparison of the computation time between the EKF, NH∞ and FANH∞ filters. As can be observed from this table, the proposed FANH∞ filter is computationally expensive compared to the EKF and NH∞ filter approaches. However, it does not affect the real-time processing of our SINS/GPS algorithm because the frequency of the FANH∞ filters is 67.56 Hz (Table 2), whereas the required frequency for the FANH∞ filter (in our case) is 10 Hz. Furthermore, the FANH∞ filter significantly improves the precision of the UAV localization process compared to the standard NH∞ filter.

**Figure 10.**
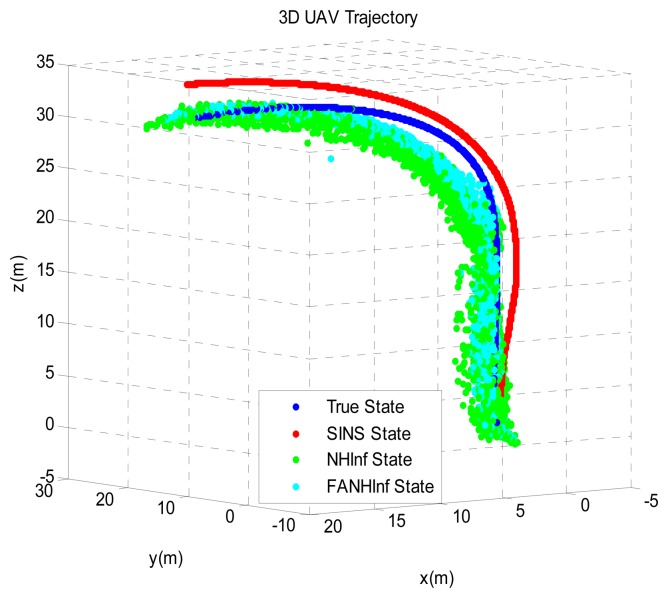
SINS/GPS 3D trajectory estimation.

**Figure 11.**
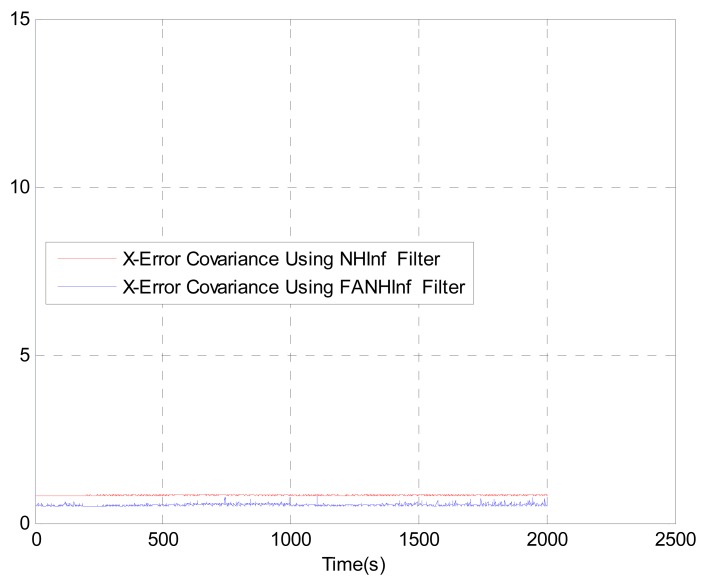
Error covariance in *x*-axes.

**Figure 12.**
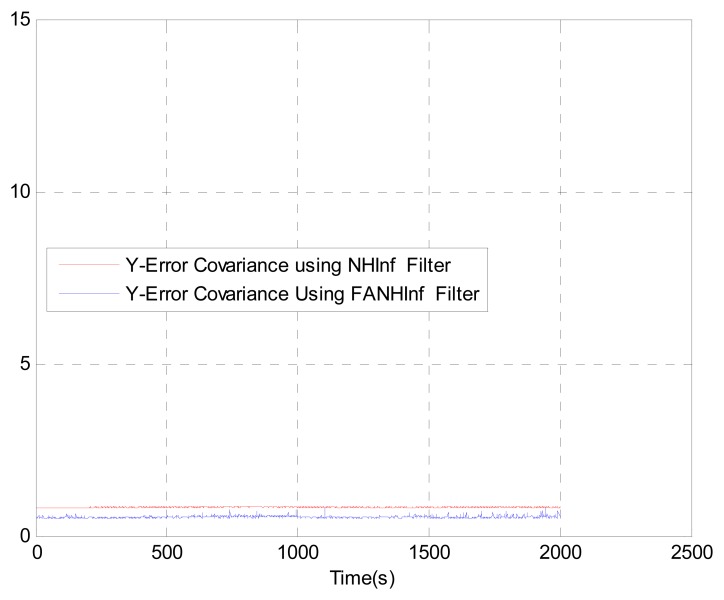
Error covariance in *y*-axes.

**Figure 13.**
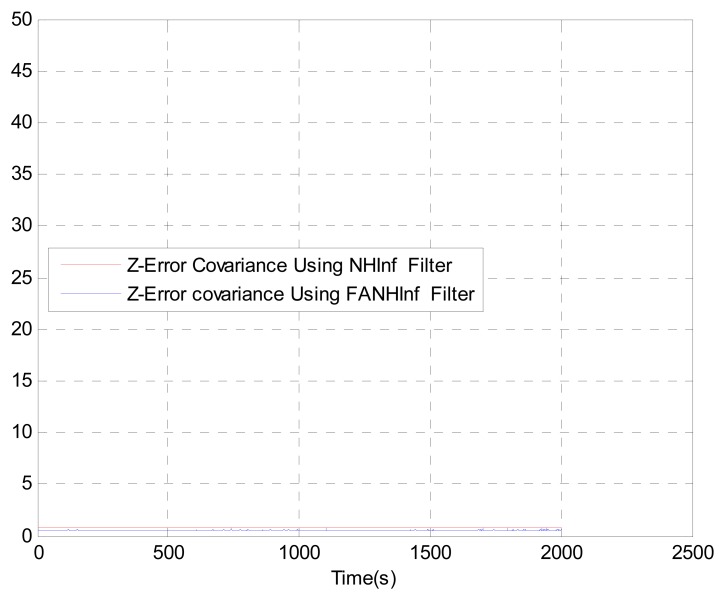
Error covariance in *z*-axes.

**Figure 14.**
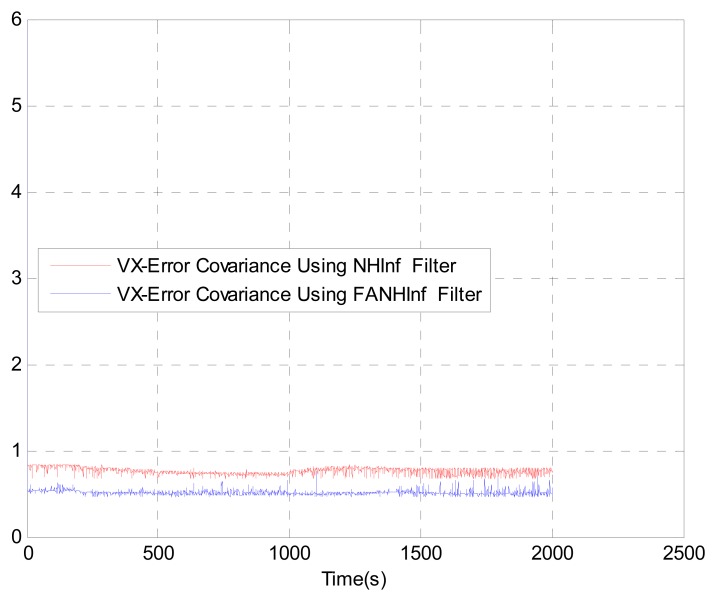
Velocity error covariance in *x*-axes.

**Figure 15.**
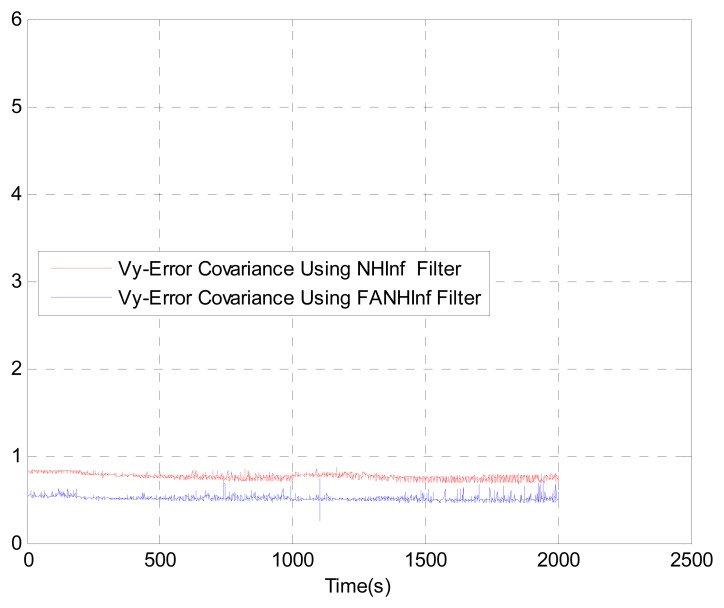
Velocity error covariance in *y*-axes.

**Figure 16.**
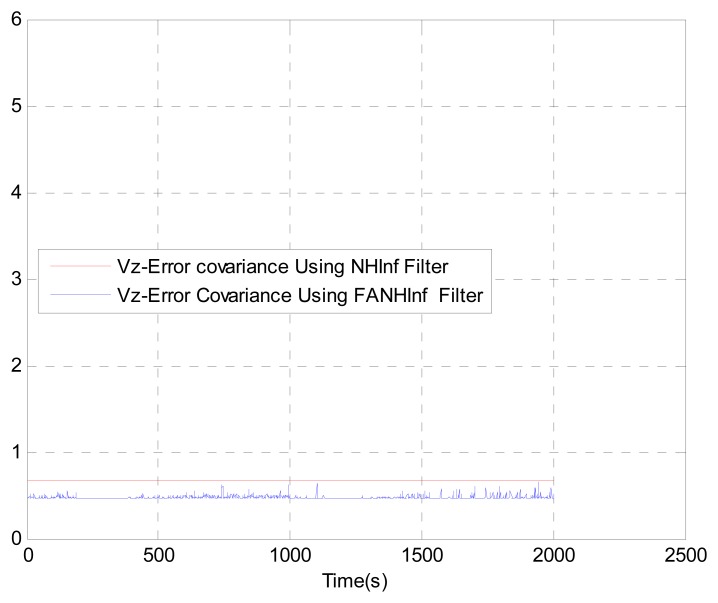
Velocity error covariance in *z*-axes.

We evaluate the computation time between the NH∞ and FANH∞ filters in Table 2 for 100 iterations because it is more significant. Figure 10 provides a comparison of the 3D trajectory of a UAV during navigation using the SINS/GPS fusion. Figures 11, 12 and 13 present a comparison of the error covariances obtained from the covariance propagation in the *x*-, *y*- and *z*-axes, respectively. It is evident that the FANH∞ filter (adaptive bounds) is more accurate than the classical NH∞ filter, where the bounds are fixed (fixed bounds). Similar results for the error covariance obtained from the covariance propagation for the UAV velocities in the *x*-, *y*- and *z*-axes are provided in Figures 14, 15 and 16, respectively.

**Figure 17.**
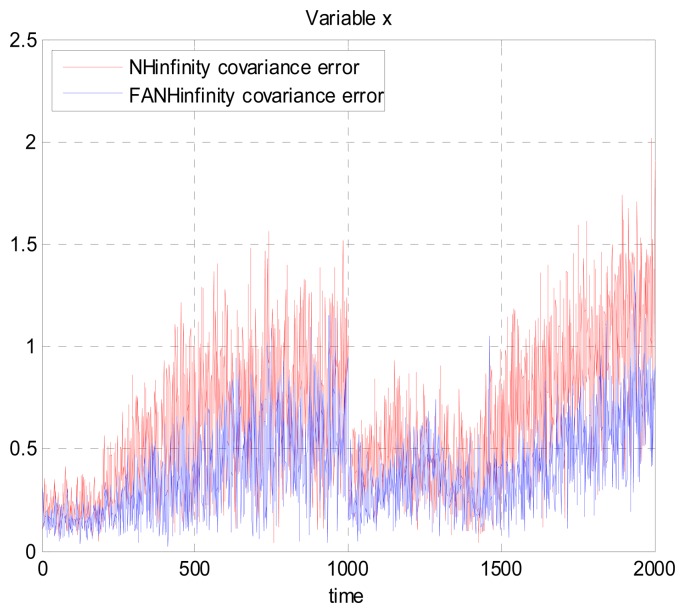
Error covariance in *x*-axes using the true error.

**Figure 18.**
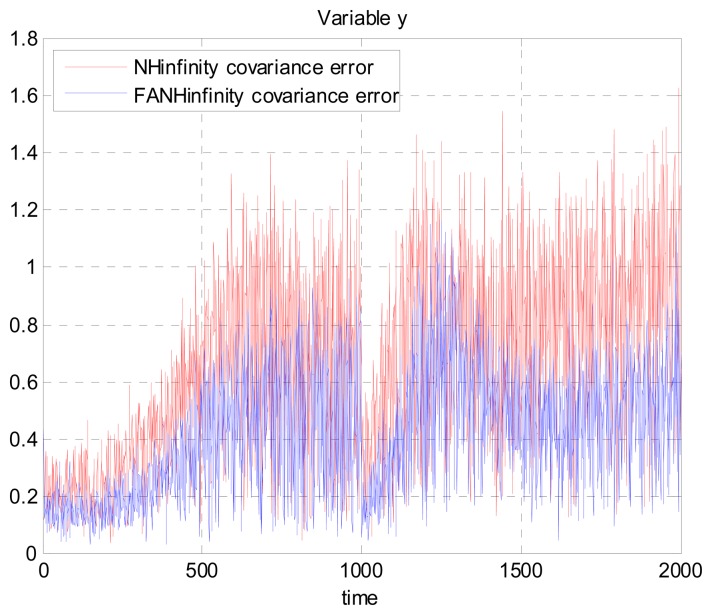
Error covariance in *y*-axes using the true error.

**Figure 19.**
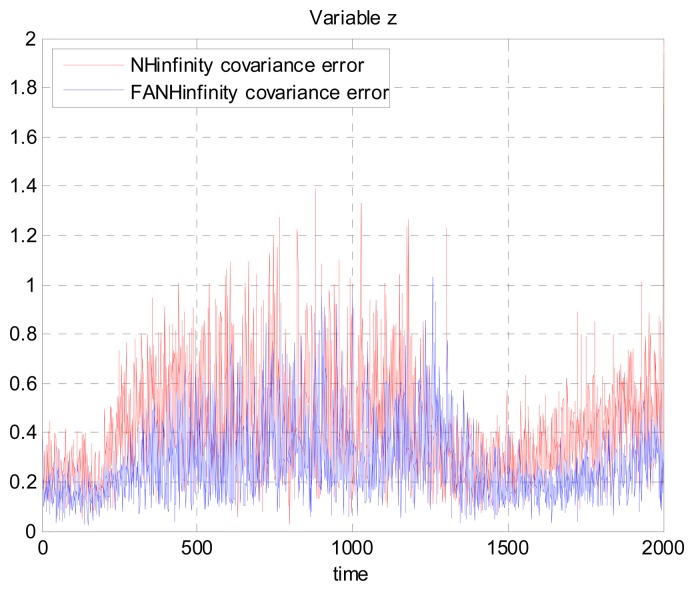
Error covariance in *z*-axes using the true error.

**Figure 20.**
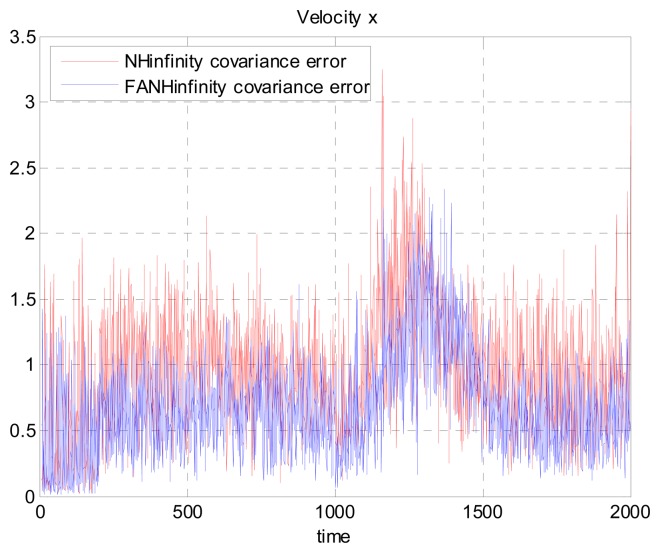
Velocity error covariance in *x*-axes using the true error.

**Figure 21.**
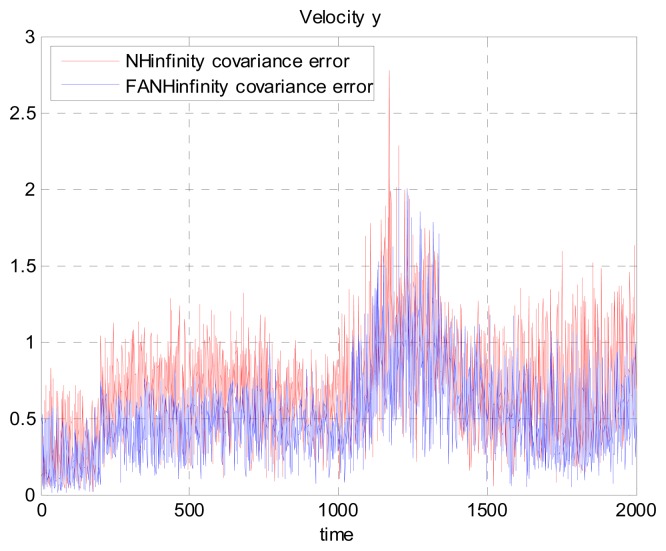
Velocity error covariance in *y*-axes using the true error.

**Figure 22.**
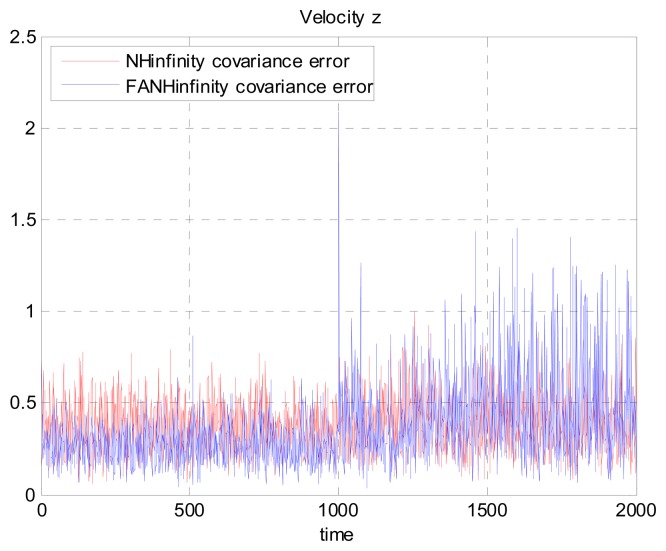
Velocity error covariance in *z*-axes using the true error.

Figures 17, 18 and 19 present a comparison of the error covariances in the *x*-, *y*- and *z*-axes, respectively, obtained from the true error. As seen from these results, the error covariances of positions obtained by the FANH∞ filter are smaller compared to those obtained by the H∞ filter. Similar results of the error covariance obtained from the true error for the UAV velocities in the *x*-, *y*- and *z*-axes are provided in Figures 20, 21 and 22, respectively.

## 7. Conclusions

In this paper, we have proposed a Fuzzy Adaptive Nonlinear H∞ filter for the SINS/GPS data fusion for UAV localization. The FANH∞ filter uses two fuzzy inference systems to adaptively tune the linearization error bounds and the H∞ norm bound. This adaptive tuning provides more robustness and consistency for the filter, which leads to results that are more accurate. The proposed approach is implemented and compared with the classical NH∞ filter using the error covariances calculated from the true errors. Satisfactory results have been obtained for the estimation of the positions and velocities, and the suitability for real-time implementation has been maintained.

## Figures and Tables

**Table 1.** Comparison of the standard deviation between the EKF, NH∞ and FANH∞ filters.

	*σ_x_*(*m*)	*σ_y_*(*m*)	*σ_z_*(*m*)
**EKF**	7.7919	25.0034	4.1430
**NH∞**	1.9677	1.8295	3.2783
**FANH∞**	0.8245	0.7659	1.3944

**Table 2.** Comparison of the computation time between the NH∞ and FANH∞ filters.

	**NH∞**	**FANH∞**
**Required time for 100 iterations (s)**	0.0294	1.4849
**Frequency (Hz)**	3401.3	67.56
